# Investigating Milk Fat Globule Structure, Size, and Functionality after Thermal Processing and Homogenization of Human Milk

**DOI:** 10.3390/foods13081242

**Published:** 2024-04-18

**Authors:** Gulustan Ozturk, Bruna Paviani, Rewa Rai, Randall C. Robinson, Sierra D. Durham, Mara I. Baller, Aidong Wang, Nitin Nitin, Daniela Barile

**Affiliations:** 1 Department of Food Science and Technology, University of California, Davis, Davis, CA 95616, USA; bpaviani@ucdavis.edu (B.P.); rnrai@ucdavis.edu (R.R.); rcrobinson@ucdavis.edu (R.C.R.); sddurham@ucdavis.edu (S.D.D.); miballer@ucdavis.edu (M.I.B.); aidwang@ucdavis.edu (A.W.); nnitin@ucdavis.edu (N.N.); 2 Department of Food Science, University of Wisconsin-Madison, Madison, WI 53706, USA; 3 Foods for Health Institute, University of California, Davis, Davis, CA 95616, USA

**Keywords:** MFGM, vat pasteurization, retort, homogenization, ultra-high temperature, sialic acid, xanthine oxidase, bioactivity

## Abstract

Human milk provides bioactive compounds such as milk fat globules (MFGs), which promote brain development, modulate the immune system, and hold antimicrobial properties. To ensure microbiological safety, donor milk banks apply heat treatments. This study compares the effects of heat treatments and homogenization on MFG’s physicochemical properties, bioactivity, and bioavailability. Vat pasteurization (Vat-PT), retort (RTR), and ultra-high temperature (UHT) were performed with or without homogenization. UHT, RTR, and homogenization increased the colloidal dispersion of globules, as indicated by increased zeta potential. The RTR treatment completely inactivated xanthine oxidase activity (a marker of MFG bioactivity), whereas UHT reduced its activity by 93%. Interestingly, Vat-PT resulted in less damage, with 28% activity retention. Sialic acid, an important compound for brain health, was unaffected by processing. Importantly, homogenization increased the in vitro lipolysis of MFG, suggesting that this treatment could increase the digestibility of MFG. In terms of color, homogenization led to higher L* values, indicating increased whiteness due to finer dispersion of the fat and casein micelles (and thus greater light scattering), whereas UHT and RTR increased b* values associated with Maillard reactions. This study highlights the nuanced effects of processing conditions on MFG properties, emphasizing the retention of native characteristics in Vat-PT-treated human milk.

## 1. Introduction

Human milk is a dynamic biological system that provides nutrients and bioactive compounds essential for infant survival, development, and health [1]. Milk fat globules (MFGs) are gaining increasing importance among bioactive compounds due to their biological and functional roles. MFG has a uniquely complex structure and composition, with a triacylglycerol core representing 98% of the total lipids found in milk and providing most of the infant’s energy intake besides the essential fatty acids required for the infant’s growth and development [2]. This triacylglycerol (TAG) core is enveloped by a three-layer natural biological membrane that originates from the mammary gland epithelial cells and is called the milk fat globule membrane (MFGM) [3]. The unique colloidal assembly is designed by evolution to be highly digestible by newborn infants, as its digestion and absorption are essential for several functions, including brain development [4]. The MFGM is mainly composed of polar lipids and glycolipids (roughly 40%) and specific membrane proteins and glycoproteins (~60%) [5,6], which are delivered in the gastrointestinal tract [7]. One important bioactive protein embedded in the MFGM is xanthine oxidase, the second most abundant protein component of the globules [8], which is known to provide antimicrobial protection to newborns [9,10]. The N-glycosylated proteins and glycolipids found on MFGM [11] are decorated with sialic acids, which have been shown to play a pivotal role in brain development and shaping the infant’s gut microbial populations [12]. 

Human milk represents the optimal nutrition for all newborns, and the World Health Organization (WHO) recommends exclusively breastfeeding infants for the first six months of life [13]. However, when human milk is unavailable or insufficient for a variety of factors, donor human milk is frequently used as the best alternative for vulnerable newborns. Access to human milk is critical, especially for preterm infants, due to their precarious health conditions. The American Academy of Pediatrics recommends pasteurized donor human milk as the second choice for feeding preterm infants [13].

Because human milk is not sterile, it needs to be heat-treated before consumption to ensure microbiological safety by inactivating viral and bacterial agents [14]. The typical heat treatments used by the dairy industry are high-temperature short-time (HTST) (72 °C for 15 s), ultra-high-temperature (UHT) (135–150 °C for 2–6 s), and retort (RTR) (121 °C, at 138 kPa for 5 min) processing [15,16] to ensure microbial safety and extend shelf life. Donor milk banks and some human milk processing companies use Vat pasteurization (Vat-PT), which employs milder temperatures for a longer time (62.5 °C for 30 min), in the hope of potentially preserving the bioactivity of compounds [17,18,19]. Others use retort to obtain a shelf-stable product [20]. In addition, dairy processing often includes homogenization, a high-pressure mechanical process that is used to reduce the size of the natural fat globules of milk by pumping milk at a high pressure (10–30 MPa) through a small valve. The resulting uniformly small particles distribute evenly in milk, thereby preventing the separation of a cream layer during storage of the final product [14]. Some newly available human-based nutritional products on the market are also homogenized and heat-treated at higher temperatures to increase their shelf life [21]. Yet, the impact of said processes on MFG’s biological activity, structure, and function remains unknown. There are several reports on the effects of various thermal processing methods, such as Vat pasteurization, HTST, retort, and microwave-assisted heating, or non-thermal processes such as ultraviolet-C irradiation and high-pressure processing (HPP). However, those are selectively focused on assessing select proteins, enzymes, oligosaccharides, and the overall composition of lipids in human milk [20,22,23,24,25,26]. A few studies in the literature also report the impact of combining thermal processing with or without homogenization techniques (e.g., ultrasonication) on the digestive behavior of human milk in vitro and in vivo [27,28]. However, it remains unclear how the processing methods used by human milk banks or human milk processing companies (such as UHT and RTR with or without homogenization) impact MFG.

In this study, the effects of Vat-PT, RTR, and UHT treatment in the presence or absence of homogenization on (a) the microstructure of milk fat globules, (b) the bioactivity and glycosylation of milk fat globules, and (c) the bioavailability of milk fat measured via in vitro hydrolysis of milk TAG by the pancreatic lipase using the indirect titration method were evaluated. In addition, the color parameters measuring L* (lightness), a* (red/greenness), and b* (yellow/blueness) were examined in all processed samples. Gaining a clear understanding of how current processing methods affect MFG is critical for optimizing processing conditions to preserve bioactivity for future product formulation.

## 2. Methods and Materials

### 2.1. Human Milk Handling, Storage, and Composition

The goal of this work was to assess industrial-scale treatments for donor milk since lab-scale tests using small volumes of milk are not representative of the impact of industrial processing techniques on key bioactive components of human milk. Since collecting fresh milk from donors locally delivered insufficient amounts, we collaborated with Prolacta Bioscience (City of Industry, CA, USA) to obtain 250 L of pooled frozen human milk, which had been collected from 32 previously screened donors. Upon receipt of the frozen pooled human milk, gentle thawing was applied, following recommendations for milk banks best practices endorsed by the American Academy of Pediatrics [29]. FTIR was used to quantify protein and fat according to AOAC 972.16 (LactoScope FTIR Mid-IR Dairy Analyzer by Delta) [30].

### 2.2. Human Milk Processing at the Industrial Scale

A total of 250 L of pooled frozen human milk was transported frozen (−20 °C) from Prolacta Bioscience (City of Industry, CA, USA) to our Milk Processing Laboratory facility at the University of California Davis.

**Figure 1:**
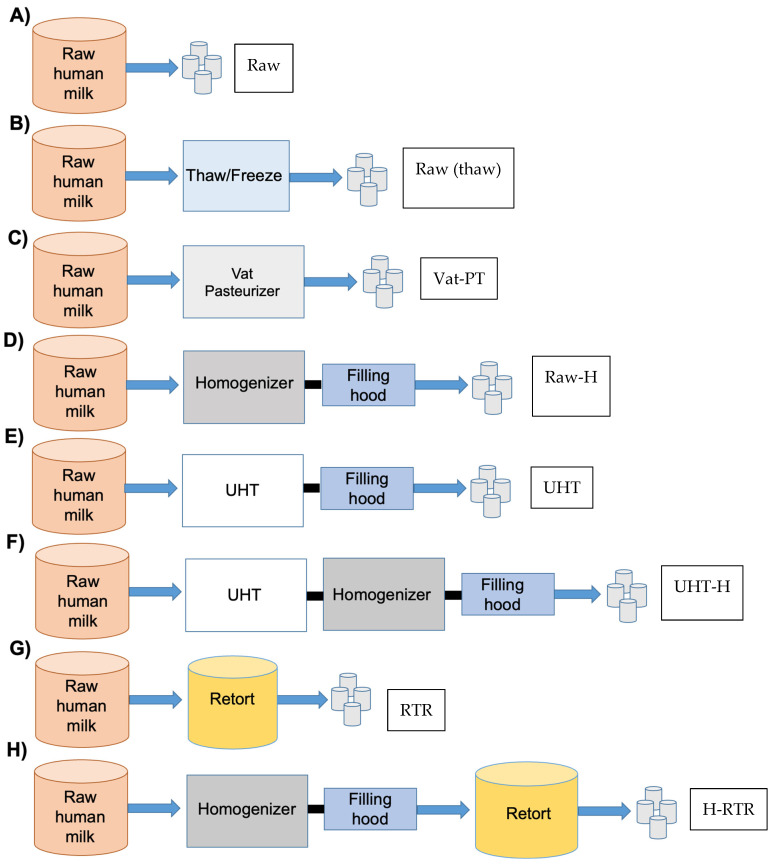
Schematic representation of the experimental design and naming convention for human milk processing. The pooled, thawed milk was divided into eight aliquots: (**A**) pooled raw milk previously frozen at −20 °C, labeled Raw, no treatment; (**B**) milk thawed at 4 °C for 72 h and refrozen at −20 °C to simulate the freeze–thaw cycles that occur in milk banks/commercial facilities, labeled Raw (thaw); (**C**) milk pasteurized by conventional Vat Pasteurizer (62.5 °C, 30 min), labeled Vat-PT; (**D**) milk homogenized (55 °C, 17 MPa) using a GEA Niro Soavi and labeled Raw-H; (**E**) milk subjected to ultra-high temperature (UHT) (142 °C, 6 s) using a MicroThermics UHT/HTST 25 EHV Hybrid Pasteurizer, labeled UHT; (**F**) milk subjected UHT (142 °C for 3 s), and then homogenized (55 °C, 17 MPa), labeled UHT-H; (**G**) milk retorted (121 °C, 138 kPa for 5 min) in sealed cans, labeled RTR using a full-water immersion retort processor (PR-I900); and (**H**) milk homogenized (55 °C, 17 MPa) and retorted (121 °C, 138 kPa for 5 min) in sealed cans, labeled H-RTR.

A schematic representation of the experimental design and naming convention is presented in Figure 1. All experiments were conducted in triplicate. The pooled thawed milk was divided into eight aliquots and subjected to various treatments, as demonstrated in Figure 1.

Aliquots of the pooled human milk, referred to as Raw, were stored at −20 °C without additional treatment (Figure 1A). The remaining aliquots of the same pooled human milk were subjected to various heat treatment and homogenization procedures, as described below:

In addition to heat treatments and homogenization, freeze–thaw cycles can occur when receiving and processing donor milk at milk banks and on an industrial scale. One aliquot was thawed, frozen, and thawed, referred to as Thawed/Freeze (Figure 1B).

One aliquot of human milk was processed at 62.5 °C for 30 min by vat pasteurization by Prolacta Bioscience (City of Industry, CA, USA) resulting in vat-pasteurized human milk referred to as Vat-PT (Figure 1C).

One aliquot of human milk was homogenized at 55 °C and 17 MPa using a GEA Niro Soavi (Colombia, MD, USA) homogenizer, referred to as Raw-H (Figure 1D).

Another aliquot was processed at 142 °C for 6 s using indirect heating through the UHT/HTST 25 EHV Hybrid Lab Pasteurizer from MicroThermics (Raleigh, NC, USA). This procedure yielded UHT-processed human milk, referred to as UHT (Figure 1E). 

Furthermore, aliquots of milk were processed in sealed cans at 121 °C and 138 kPa for 5 min using a full-water immersion retort processor (PR-I900) manufactured by Stock America Inc. (Garner, NC, USA). This process resulted in retort-processed human milk, referred to as RTR (Figure 1G).

One aliquot of human milk underwent UHT treatment (142 °C for 3 s) followed by homogenization at 55 °C and 17 MPa using a GEA Niro Soavi homogenizer, referred to as UHT-H (Figure 1F). 

Another aliquot was homogenized under the same conditions and subsequently treated with RTR sterilization, referred to as H-RTR (Figure 1H).

All experiments were conducted in triplicate. After processing, milk fat globule size distribution, zeta potential, the microstructure of milk fat globules, color parameters, and xanthine oxidase activity assays were conducted immediately on the same day of processing. Each sample’s aliquots were stored at −20 °C for SDS-PAGE, sialic acid quantification, and in vitro hydrolysis of milk fat globules by pancreatic lipase.

### 2.3. Milk Fat Globule Size Distribution 

The fat globule size distribution was measured using a Microtrac S3500 (Microtrac, Montgomeryville, PA, USA) with laser light scattering. An optimized sample preparation procedure was used to determine the size distribution of MFG in human milk samples [31]. The frozen milk samples were gently thawed at 4 °C for 3 days according to [32], processed, and the size of the MFG was measured immediately. All milk samples were diluted with Milli-Q water at 9:1 water to milk (*v*/*v*), and the cream was separated via centrifugation at 3000× *g* for 5 min at room temperature. The collected cream was reconstituted in water at 4% (*w*/*v*) and centrifuged at 3000× *g* for 5 min at room temperature. The cream was then collected and reconstituted in water at 20% (*w*/*v*) concentration [32]. After that, 35 mM EDTA (pH 7.0) was added at a 1:5 (*v*/*v*) ratio. EDTA was added to the samples as a calcium chelator to dissociate casein micelles and eliminate their potential interference with MFG particle size distribution measurements. All particle size distribution measurements were conducted at room temperature. Deionized water with a refractive index of 1.33 was used as a dispersant. The dispersed phase, milk fat, was measured with a refractive index of 1.46 [31].

### 2.4. Zeta Potential of Milk Fat Globules

The zeta potential of MFG was measured by observing the electrophoretic mobility of the globules. The electrophoretic mobility is measured using a combination of by a laser Doppler velocimetry and phase analysis of light-scattering in a Malvern Zetasizer Nano ZS instrument (Malvern Instruments Ltd., Worcestershire, UK). Milk samples were diluted with Milli-Q water in a 1:9 (*v*/*v*) ratio. 

### 2.5. Microstructure of Milk Fat Globules 

The microstructural analysis of human milk was performed using an inverted Olympus IX 71 microscope (Olympus Inc., Center Valley, PA, USA) equipped with a 40×/1.3 oil objective. To collect fluorescence images, milk phospholipids in the globule membrane surrounding native milk fat globules were stained using a dye-labeled phospholipid, 1,2-Dihexadecanoyl-sn-Glycero-3-Phosphoethanolamine (DHPE), where the head group is labeled Oregon Green™ 488. The dye stock was prepared in chloroform (1 mg/mL). The MFGM was labeled with Oregon Green^®^ 488 DHPE at a final 1 μg/mL concentration. After adding the fluorescent probes, the samples were kept at room temperature for at least 15 min before the microstructural analysis. Then, 5 μL of the samples stained with the fluorescent probes were deposed onto the glass slides and observed using the microscope. The microstructural analyses were performed at room temperature. The fluorescence images were collected with an excitation wavelength of 488 nm, and the emission signal was collected in the FITC region (515–550 nm). 

### 2.6. Color Parameters

The color parameters were determined using a Hunter colorimeter (Hunter Associates Laboratory, Reston, VA, USA) with a CIELab scale (L*, a*, and b*). The color values L* (lightness), a* (red/greenness), and b* (yellow/blueness) of milk samples were measured using L* values (0–100) representing black to white, a* values representing green to red, and b* values representing blue to yellow. The colorimeter was calibrated using a white tile and a black tile [33,34].

### 2.7. Xanthine Oxidase Activity 

The activity of xanthine oxidase in human milk samples was measured using Amplex™ Red Xanthine/Xanthine Oxidase Assay Kit (A22182, Invitrogen, Waltham, MA, USA), according to the manufacturer’s instructions. Milk samples were diluted 2× in reaction buffer, mixed with Amplex red, horseradish peroxidase, xanthine, and reaction buffer, and incubated for 30 min at 37 °C. The production of H_2_O_2_ from the oxidation of xanthine to uric acid was measured fluorometrically using an M5 plate reader (Molecular Devices, San Jose, CA, USA). H_2_O_2_, in the presence of horseradish peroxidase, reacts stoichiometrically with the Amplex™ Red reagent to generate the red-fluorescent oxidation product, resorufin. Resorufin has fluorescence excitation in the 530–560 nm range and emission at 590 nm. 

### 2.8. Distribution of MFGM Proteins in Fractions of Homogenized and Non-Homogenized Raw Human Milk by SDS-PAGE

Homogenized and non-homogenized raw human milk samples were centrifuged at 3000× *g* for 5 min at room temperature to separate the skim and cream fractions and compare them with the whole milk sample. Sodium dodecyl sulfate-polyacrylamide gel electrophoresis (SDS-PAGE) analysis was performed to visualize the presence of MFGM-associated proteins in whole milk, skim milk, and cream samples before and after homogenization. All samples were diluted ten times in water, and then the same volume of each sample was combined with a Laemmli sample buffer and 0.2 M dithiothreitol (DTT). Samples were heated at 95 °C for 5 min. Each sample mixture was then loaded onto a 4–15% acrylamide gel (Bio-Rad Laboratories, Hercules, CA, USA), and the proteins were separated at 185 V. Precision Plus Protein Dual Color Standards mixture (Bio-Rad) was used as a positive control. The gel was stained with Coomassie blue (Bio-Rad) and de-stained with water.

### 2.9. Sialic Acid Quantification 

The concentration of total, free, and MFGM-associated sialic acid (N-acetylneuraminic acid) in human milk was measured according to the methods of Hurum and Rohrer [35]. For measuring the total sialic acid, whole human milk samples were mixed with 100 mM of sulfuric acid in a 1:1 ratio, incubated at 80 °C for 1 h to hydrolyze the bound sialic acid, and centrifuged at 2000× *g* and 5 °C for 10 min to collect the solids. The supernatants containing the sialic acid were purified with Dionex OnGuard II A 1 mL solid phase extraction cartridges (ThermoFisher Scientific, Waltham, MA, USA). The cartridges were conditioned with 10 mL of Milli-Q water before sample loading. After loading the samples, the cartridges were washed with an additional 10 mL of Milli-Q water, and sialic acid was eluted with 12 mL of 50 mM NaCl. The samples were dried and reconstituted in Milli-Q water. Total sialic acid was quantified on the milk samples using a high-performance anion-exchange chromatography system with pulsed amperometric detection (HPAEC-PAD) (Thermo Scientific Dionex ICS-5000+), equipped with an electrochemical cell with a disposable gold working electrode and a pH-Ag/AgCl reference electrode. Chromatographic separation of sialic acid was carried out with a Dionex CarboPac PA20 analytical column (3 × 150 mm, Thermo Scientific) and a Dionex CarboPac PA20 guard column (3 × 30 mm, Thermo Scientific), with a solvent flow of 0.5 mL/min. Eluents consisted of (A) water, (B) 200 mM sodium hydroxide in water, and (C) 500 mM sodium acetate in water. Samples were analyzed using a 20 min gradient with eluent B held isocratic at 50%. Eluent C was increased from 2 to 40% between 0 and 15 min, then held constant at 40% from 15 to 20 min. The column temperature was kept at 30 °C.

The concentration of free sialic acid, naturally occurring at trace levels in milk, was measured directly in the same human milk samples before hydrolysis of the bound sialic acid. Samples were prepared and analyzed as described above, except for the sulfuric acid hydrolysis.

The contribution of MFGM-associated sialic acid to the total sialic acid was calculated as the difference between the corresponding total and free sialic acid concentrations. All samples for sialic acid analysis were extracted and analyzed in duplicate.

### 2.10. Lipolysis of Milk Fat Globules by the Pancreatic Lipase

Pancreatic lipase (porcine pancreatic extract (pancreatin)) (#P7545; 8 × USP specification), sodium taurodeoxycholate (NaTDC), glyceryl tributyrate (tributyrin, ≥99%; #T8626), and Trisaminomethane (Tris) were purchased from Sigma Aldrich. The dispersion of the enzyme powder (1 mg/mL) was prepared in cold MilliQ water and kept on ice until the assay was performed. Pancreatic lipase activity was measured according to Grundy et al. [36]. In short, the assay solution contained 0.3 mM of Tris, 150 mM of NaCl, 2 mM of CaCl_2_, and 4 mM of NaTDC. The substrate emulsion was prepared at 37 °C while mixing 14.5 mL of the assay solution and 0.5 mL of tributyrin (the substrate for the pancreatic lipase). After the temperature was stabilized, the pH was adjusted to 8. Then, 50 µL, 100 µL, or 200 µL of the enzyme stock solution was added, and NaOH (0.1 N) delivery was recorded immediately as a function of time for 5 min. After ensuring a proper level of pancreatic lipase enzyme activity (65 U/mg), which agreed with literature values [36], the degree of lipolysis was measured indirectly in milk samples. Milk samples (0.5 mL) were dispersed in the 14.5 mL of assay solution. After the temperature was stabilized, the pH was adjusted to 8. 200 µL of the enzyme stock solution was added, and the amount of 0.1 N NaOH required to maintain equilibrium for 20 min was recorded for each sample, modified from [37,38].

## 3. Statistical Analysis

One-way analysis of variance (ANOVA) with post hoc Tukey’s tests were used to identify significant (*p* < 0.05) or (*p* < 0.0001) differences among samples (Graph Pad Prism, version 8.2). The results are shown as the average ± standard deviation.

## 4. Results 

### 4.1. Human Milk Composition

**Figure 2:**
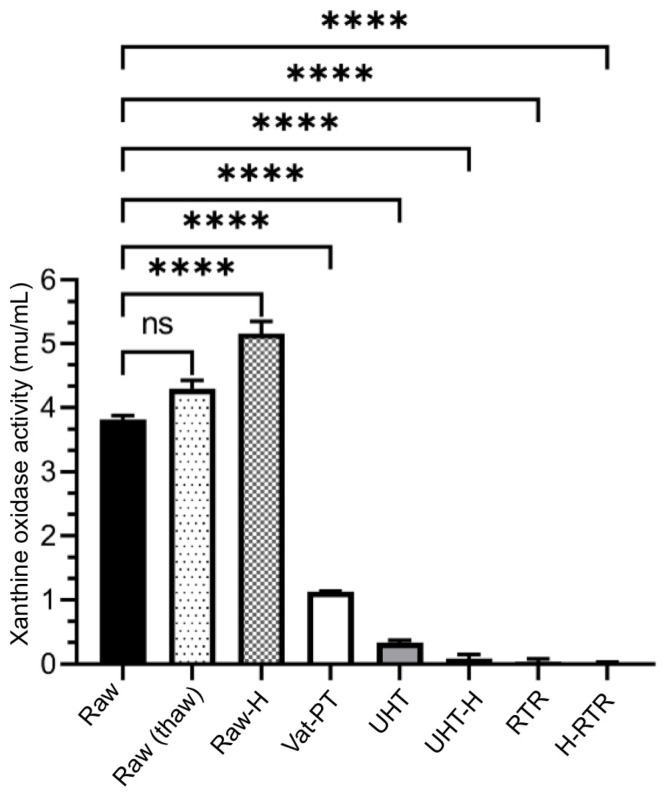
Xanthine oxidase activity in milk samples including Raw, Raw (thaw), Raw-H, Vat-PT, UHT, UHT-H, RTR, and H-RTR. One-way analysis of variance (ANOVA) with post hoc Tukey’s tests were used to identify significant differences between treatments, denoted by stars. ns: not significant, ****: *p* ≤ 0.0001.

The average fat and protein contents in the human milk used in this study were 4.55% and 1.00%, respectively. The mean diameter of raw milk MFG was 13.55 ± 0.1 µM, and the zeta potential was −16.00 ± 0.50 mV (Table 1). Raw human milk was used as a control for all experiments and exhibited an average activity of 3.81 ± 0.3 mU/mL for xanthine oxidase (Figure 2). The total, free, and MFGM-associated sialic acid concentrations in raw human milk were 0.2400 ± 0.0200 g/L, 0.0022 ± 0.0000 g/L, and 0.0900 ± 0.0040 g/L, respectively.

### 4.2. Changes in MFG Particle Size Distribution, Zeta Potential, and Membrane Induced by Homogenization and Thermal Processing

**Figure 3:**
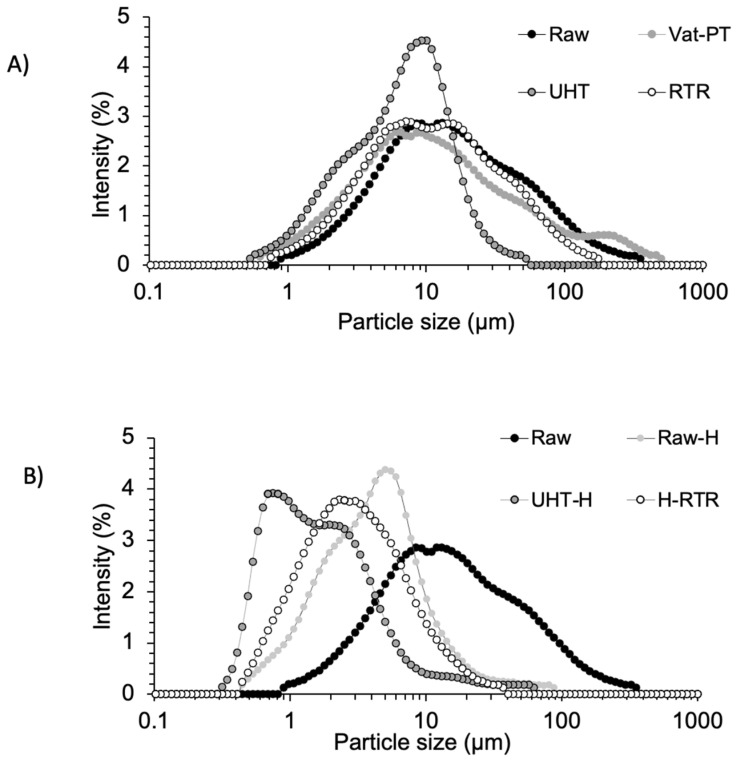
Particle size distributions of the milk samples measured by laser light scattering, including (**A**) Raw, Vat-PT, UHT, and RTR; (**B**) Raw, Raw-H, UHT-H, and H-RTR milk samples.

Figure 3A,B compare the MFG size distribution for the selected thermal and mechanical processing methods, respectively. The average size distribution of MFG after Vat-PT and RTR was similar to that of raw human milk (untreated). When comparing homogenized samples with raw human milk, the curve shifted toward smaller values after homogenization. Table 1 presents the physicochemical characteristics (average diameter (µM) and zeta potential (mV) values) of MFG in all milks heat-treated with or without homogenization. The average diameter of MFG after Vat-PT and RTR processing was similar to raw human milk; however, the average diameter of MFG was reduced by UHT. Homogenization of raw human milk and heat-treated milk decreased the size of the MFG by 2 to 4-fold. Heat treatments employing temperatures at 142 °C and homogenization increased the absolute values of the zeta potential of MFG compared to raw human milk and Vat-PT.

**Figure 4:**
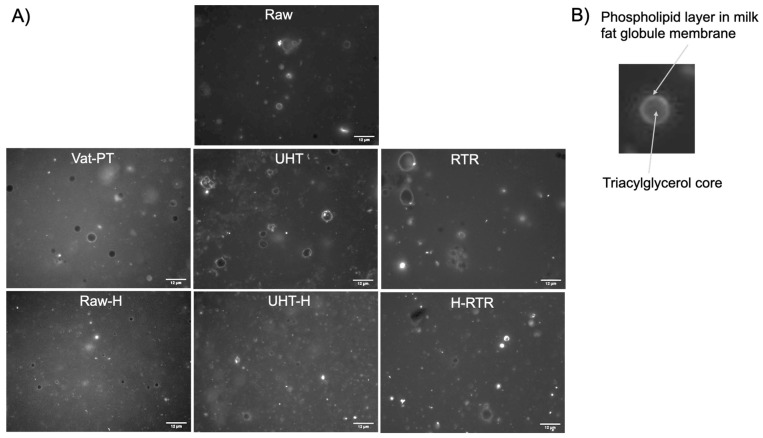
Fluorescence microscopy images (×40 objective) showing the phospholipid layer at the surface of MFG in select processed samples and the control (raw). (**A**) Raw, Vat-PT, UHT, RTR, Raw-H, UHT-H, and H-RTR. (**B**) Zoomed-in image of a milk fat globule from raw milk showing phospholipids in the biological membrane and the MFGM surrounding the triacylglycerol core of MFG. Phospholipids were stained with Oregon Green™ 488. Scale = 12 μm.

**Figure 5:**
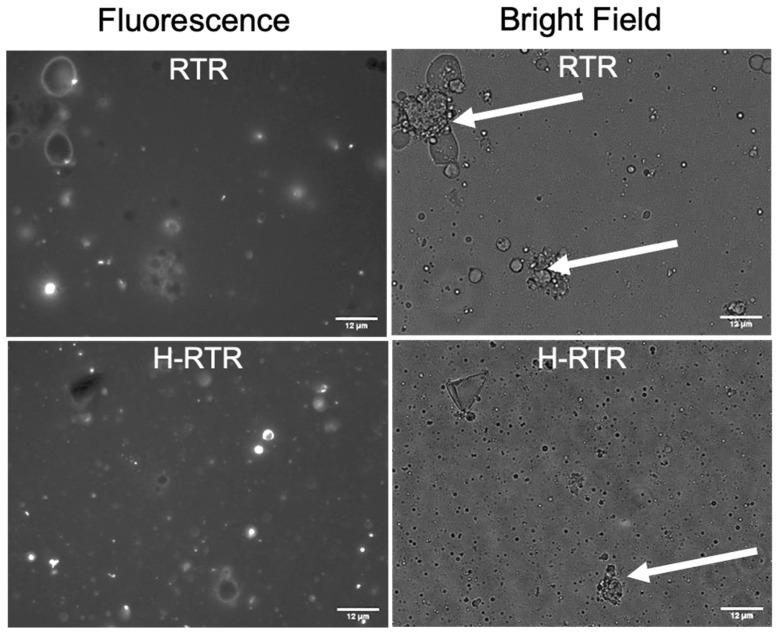
Fluorescence microscopy images (×40 objective) and bright-field images of RTR and H-RTR processed samples. Phospholipids were stained with Oregon Green™ 488. White arrows are pointing at heat-induced protein aggregates in milk. Scale = 12 μm.

To investigate the changes in the organization of the phospholipid layer at the surface of MFG after processing, the MFGM was labeled with a membrane-specific dye (Oregon Green™ 488 DHPE dye-labeled phospholipid). The images presented in Figure 4 demonstrate that the phospholipid layer was maintained on the surface of raw milk globules (Figure 4A—raw human milk; Figure 4B—a zoomed-in image of raw human milk). The intact ring-shaped phospholipid layers were seen at the surface of milk fat globules in all samples, suggesting that the integrity of the MFGM was maintained after thermal processing and homogenization. The additional observation of the same samples under bright-field microscopy indicated that the RTR samples led to heat-induced protein aggregates (indicated by white arrows in Figure 5). Protein aggregates in RTR samples were more frequently seen and larger than those in UHT and UHT+H (Supplementary Figure S1). Although RTR processing caused the formation of aggregates, homogenization successfully decreased the size of the aggregates (Figure 5, bright-field images of H-RTR samples). In addition, we observed fluorescence debris in the microscopic images of the RTR samples compared to the raw milk, suggesting the formation of smaller lipid particles in addition to aggregates.

### 4.3. Xanthine Oxidase Activity

The effect of Vat-PT, UHT, RTR, and homogenization on xanthine oxidase activity was determined using xanthine as the substrate for a specific enzymatic reaction. Xanthine oxidase activity decreased by 72% after Vat-PT (Figure 2), whereas it was completely lost after RTR processing, and a 93% decrease was seen for UHT compared to raw human milk. On the other hand, homogenization increased the availability of xanthine oxidase to its substrate (xanthine), so xanthine oxidase activity was significantly higher in homogenized raw human milk (38%). Xanthine oxidase activity was additionally measured in samples that underwent one freeze–thaw cycle and did not change significantly with this treatment. 

### 4.4. Distribution of MFGM Proteins in Fractions of Homogenized and Non-Homogenized Human Milk

**Figure 6:**
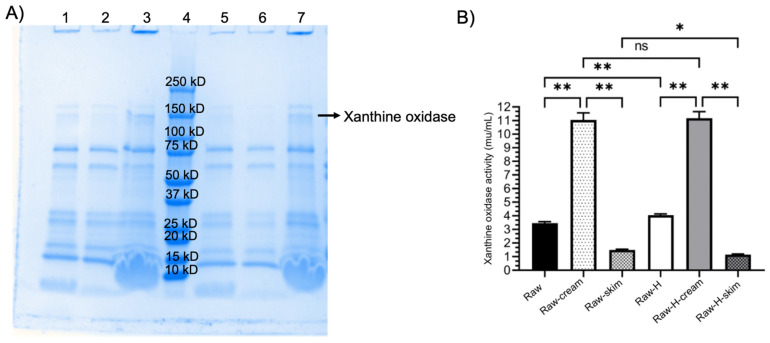
Panel (**A**). SDS-PAGE profile of raw human milk and raw homogenized human milk and their fractions; lane 1 (Raw), lane 2 (Raw skim), lane 3 (Raw cream), lane 4 (protein standards), lane 5 (Raw-H), lane 6 (Raw-H skim), and lane 7 (Raw-H cream). Panel (**B**). Xanthine oxidase activity in raw whole human milk and homogenized whole human milk and their fractions (Raw, Raw cream, Raw skim, Raw-H, Raw-H cream, and Raw-H skim). The same volume of each sample was loaded. One-way analysis of variance (ANOVA) with post hoc Tukey’s tests were used to identify significant differences between treatments, denoted by stars. ns: not significant *: *p* ≤ 0.05, **: *p* ≤ 0.01.

The protein profile of the homogenized raw human milk/cream sample displayed in Figure 6A was closer to the non-homogenized raw human milk/cream sample than the whole milk and skim fractions. Similarly, bands corresponding to xanthine oxidase (140 kDa) appeared in a more intense color and thickness for the cream fraction, indicating that xanthine oxidase was not released from MFGM after homogenization but was enriched in the cream by the centrifugation process. To gain further confirmation, we also measured xanthine oxidase activity in all samples and demonstrated that the activity in the cream was significantly higher than that in skim milk (Figure 6B).

### 4.5. Sialic Acid

**Figure 7:**
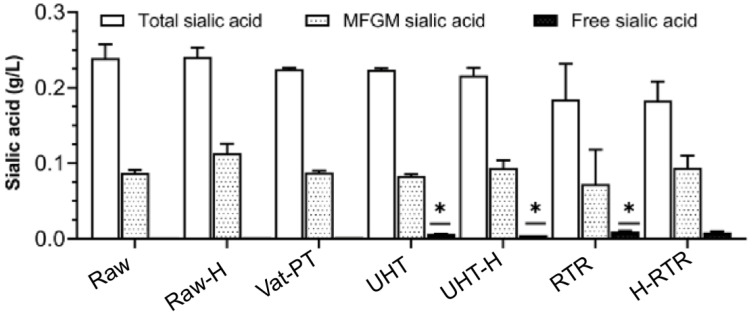
The total, MFGM-associated, and free sialic acid concentrations in milk samples, including Raw, Raw-H, Vat-PT, UHT, UHT-H, RTR, and H-RTR. One-way analysis of variance (ANOVA) with post hoc Tukey’s test was used to identify significant differences between treatments, denoted by stars. * *p* ≤ 0.05.

No statistically significant changes were observed in the content of total and MFGM-associated sialic acid when raw human milk was processed. However, substantial variability among replicates was observed for the RTR-processed samples (Figure 7). Free sialic acid was significantly increased in UHT, UHT-H, and RTR samples (*p* < 0.05), whereas the vat-pasteurized samples presented values close to the unprocessed samples (Supplementary Figure S2).

### 4.6. Color Parameters

The results for Hunter’s parameters L* (lightness), a* (red/greenness), and b* (yellow/blueness) of the donor milk samples are shown in Table 2. Homogenization led to higher L* values due to the finer dispersion of the fat, whereas aggressive thermal processes increased b* values, an indicator of Maillard reactions.

### 4.7. Lipolysis of Milk Fat Globules by the Pancreatic Lipase

**Figure 8:**
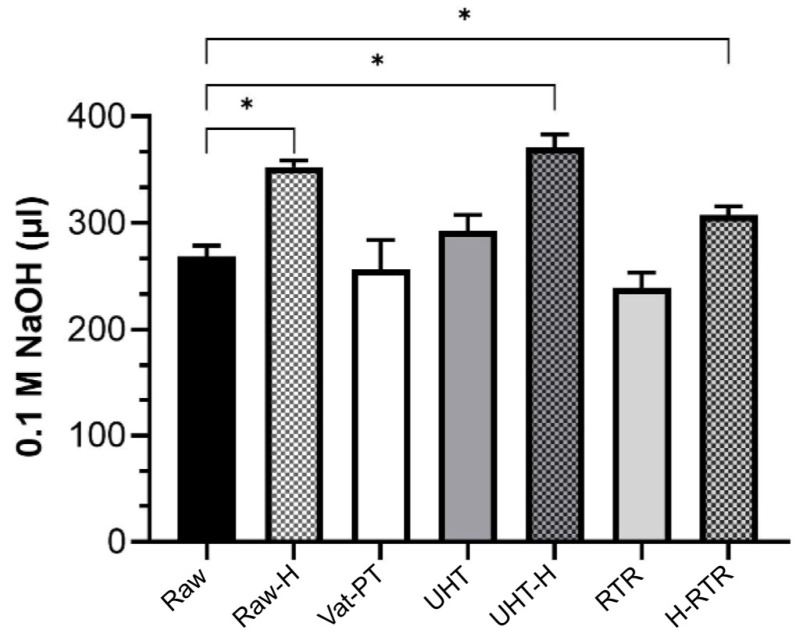
Lipolysis of milk fat globules by the pancreatic lipase. Titration of free fatty acids during the simulated digestion at pH 8 over 20 min with pancreatic lipase using raw milk samples including Raw, Raw-H, Vat-PT, UHT, UHT-H, RTR, and H-RTR. One-way analysis of variance (ANOVA) with post hoc Tukey’s tests were used to identify significant differences between treatments, denoted by star. * *p* ≤ 0.05.

Lipolysis was assessed indirectly by the amount of NaOH consumed by titrate-free fatty acids over time in all samples (Figure 8). The amount of NaOH consumed by titrate-free fatty acids was significantly higher in the homogenized samples. Homogenization significantly increased MFG’s lipolysis (and therefore its digestibility) in human milk.

## 5. Discussion

Human milk and human milk-based products with various compositions and shelf lives are available on the market. However, the extent to which different processing techniques affect the structure, activity, and bioavailability of milk fat globules in these products remains unknown. Here, the effects of thermal processing techniques (Vat-PT, UHT, and RTR) with or without homogenization on the microstructure (size, zeta potential, and distribution of MFGM), bioactivity markers (xanthine oxidase activity and sialic acid), and bioavailability of milk fat (in vitro hydrolysis of milk fat globules by pancreatic lipase) were investigated.

It is well known that homogenization reduces the mean diameter of the MFG [39], and the values obtained here agree with the literature. The mean diameter of raw milk MFG (13.55 ± 0.1 µM), which was previously frozen and thawed multiple times, does not correspond to the mean diameter of fresh human milk MFG (~4–5 µM) [40,41]; however, it is in agreement with the mean diameter of frozen and thawed human milk MFG (13.4 ± 0.2 µM) [42]. This observation implies that the repeated freeze– thaw cycles impact the mean diameter of MFG. The mean diameter of MFG in vat-pasteurized human milk (9.79 ± 0.38 µM) was similar to its unprocessed counterpart (13.55 ± 0.1 µM); however, we noticed that UHT (7.13 ± 0.34 µM) slightly reduced the mean diameter of MFG (Table 1). A similar result has been previously reported [41], with the mean diameter in Holder pasteurized human milk (12.5 ± 2.3 µM) being similar to raw milk (13.4 ± 0.2 µM), but slightly reduced with HTST (9.4 ± 1.4 µM). One possible cause of these observations could be that the UHT treatment can create structural and morphological changes in the fat and MFGM, resulting in changes in both the size and composition of the MFG [43]. Similarly, when applying RTR, the harsher heating treatment may cause significant changes in the composition and structure of the MFGM, such as denaturation and the release of proteins. These compositional changes may lead to different non-specific complexations and facilitate self-aggregations [44], as visualized in the bright-field images of RTR samples (Figure 5). In our recent collaborative study, which assessed the thermal stability of human milk proteins subjected to the same processing treatments, we observed the disappearance of bands in SDS-PAGE gels with these treatments, and aggregation was confirmed for select proteins through proteomic analysis [45,46]. In addition, the zeta potential of MFG in milk samples was measured to investigate the stability of MFG after various processes. We observed that the zeta potential of homogenized milk fat globules became more negative, leading to the increased colloidal dispersion of globules and, thus, the stability of the emulsion (Table 1). Our results agree with previous findings that homogenization increases the stability of the emulsion both by decreasing the size and increasing the net surface charge of MFG [14]. Some aggregation of MFG was observed in RTR samples, increasing the MFG size distribution (Figure 3). This result also agrees with the literature that heat treatment destabilizes the emulsion and leads to self-aggregation [44].

Xanthine oxidase activity was measured as a biomarker of the bioactivity of MFG in human milk samples. Xanthine oxidase activity was significantly higher in homogenized raw human milk than in unprocessed raw human milk. These results were expected since homogenization increases the surface area of MFG and, thus, the availability of xanthine oxidase to its substrate. Xanthine oxidase activity did not change significantly after a freeze–thaw cycle, a typical process in human milk banks and processing companies during milk collection. However, the RTR treatment completely destroyed the xanthine oxidase activity, and the UHT treatment also resulted in a 93% loss in activity (Figure 2). In contrast, the present study found that Vat-PT decreased xanthine oxidase activity by 72%, retaining 28% activity. These results suggest that vat-pasteurized milk would still provide some protection in terms of xanthine oxidase activity compared to RTR and UHT-processed milk. 

SDS-PAGE analysis was used to visualize the presence of MFGM-associated proteins, particularly xanthine oxidase, in both homogenized and non-homogenized samples to determine whether these proteins were released through homogenization. This analysis was conducted on fractionated samples to observe the impact on skim milk and cream. Both homogenized and non-homogenized samples had a more intense xanthine oxidase band in the cream fraction compared to the skim milk (Figure 6A). These results are consistent with a recent study that used SDS-PAGE analysis and xanthine oxidase as a marker to examine the effects of microfiltration and centrifugal separation on MFGM proteins and found that the intensity of xanthine oxidase bands was increased by processing for both microfiltered and separated cream in bovine milk [47]. To further confirm these findings, xanthine oxidase activity was also measured in the same set of samples used for the SDS-PAGE analysis (Figure 6B). Xanthine oxidase activity in the cream fraction was significantly higher than that in skim milk, suggesting that homogenization does not alter the localization of xanthine oxidase in the MFGM [39]. These findings are essential for future product development to fractionate MFG from homogenized or non-homogenized milk samples.

Sialic acids rarely occur in their free form in human milk, as they are more commonly present as components of oligosaccharide chains of mucins, glycoproteins, and glycolipids. This study used sialic acid as a marker of MFG glycosylation. Total and MFGM-associated sialic acid content was unaffected by heat treatments; however, free sialic acid was found to have a small (but statistically significant) increase in UHT and RTR samples, indicating a minor release of bound sialic acid. The present study allowed us to compare not only the concentrations of total sialic acid in human milk but also the form in which sialic acid was present, i.e., its distribution between total, MFGM-associated, and free forms (Figure 7, Supplementary Figure S2). Other examples in the literature validate the robustness of glycans in relation to thermal treatments. A recent study investigated the effect of microwave-assisted heating and low-temperature, long-term pasteurization on donor human milk oligosaccharides. It demonstrated that both the total amount and individual oligosaccharide concentrations were not significantly affected [24]. Another study showed that the concentration and pattern of human milk oligosaccharides were not affected by holder pasteurization in donor human milk [22]. 

Appearance attributes such as color are known to be critical factors influencing consumer purchasing decisions. Additionally, technological processes can result in color changes in dairy products, and the color of a milk product can indicate physicochemical changes. In this study, homogenization contributed to a desirably increased lightness. In contrast, the RTR process resulted in increased b* values and a darker color, an indicator of the Maillard reaction between reducing carbohydrates and amino acids, which is known for decreasing the nutritional qualities of milk and could be particularly critical for premature infants [48] (Table 2).

Milk fat globules are natural colloidal structures that aim to deliver lipids to the gastrointestinal tract of newborns, and their digestion and absorption are essential for infant development. The polar lipids within MFGM feature negatively charged anions, facilitating binding to lipase via electrostatic interactions [49]. Technological processes can affect MFG’s size and interfacial properties, with smaller globules in homogenized milk having an increased surface area, thereby providing more opportunities for the enzyme lipase to interact during gastrointestinal digestion and ultimately improving digestibility, as observed in [50]. The lipolysis of human milk samples by pancreatic lipase was indirectly measured in vitro by continuous titration of ionized free fatty acids with 0.1 M NaOH. The lipolysis rate of heat-treated samples was not significantly different from raw human milk. In contrast, the rate over time was significantly higher in the homogenized samples, including Raw-H, UHT-H, and H-RTR milk samples (Figure 8). The present study is the first to compare the effects of homogenization with or without heat treatments on the lipolysis of human milk. Existing literature demonstrating the in vitro digestion of human milk, however, is limited to non-homogenized human milk. For example, an earlier study investigating the impact of ultrasonic homogenization using a sonicator in human milk showed that ultrasonication-based homogenization increased gastric lipolysis levels in preterm infants [27]. Several studies investigate the effects of homogenization and heat treatment on bovine milk. For instance, Zhao et al. (2019) demonstrated that homogenization significantly increased the amount and initial rate of released free fatty acids, and therefore, the lipolysis of bovine MFG, thus improving MFG digestibility [50]. Berton et al. (2012) investigated the effect of homogenization on raw bovine milk fat globules and then assessed lipolysis by the human pancreatic lipase with colipase and bile salts [37]. They demonstrated that the membrane around milk fat globules is important in enhancing the mechanisms of milk lipid digestion. Similarly, in our study, we labeled MFGM with a membrane-specific dye (Oregon Green™ 488 DHPE dye-labeled phospholipid) and investigated the integrity of the membrane by fluorescence microscopy (Figure 3A), demonstrating that the phospholipid layer was maintained on the surface of globules after both thermal processing and homogenization. 

Another beneficial effect of homogenization toward lipid digestibility is related to its ability to decrease the layer of protein aggregates that were found on the surface or fat globule surface in non-homogenized RTR samples, as observed by means of bright-field microscopy (Figure 4), which likely reduced enzyme accessibility. Homogenization applied in conjunction with thermal processes reduced the undesirable aggregates and restored optimal lipolysis (Figure 8). The presence of negatively charged anions in the polar lipids within MFGM facilitates their binding to lipase via electrostatic interactions [49]. Conversely, lipid droplets coated with whey and casein proteins form a dense and thick interfacial layer, thereby reducing the availability of effective binding sites for lipase [51,52,53]. For example, Pan et al. (2024) investigated how the addition of milk fat globule membrane (MFGM) to infant formula impacts the simulated in vitro infant digestion. They demonstrated that lipid droplet surfaces coated with MFGM phospholipids resulted in free fatty acid release rates similar to those of human milk. In contrast, if the lipid droplets were encapsulated by milk proteins, they had significantly lower free fatty acid release than that of human milk or lipid droplet surfaces coated only with MFGM phospholipids [51]. 

## 6. Conclusions

This study elucidates the intricate influence of processing conditions on the properties of MFG. The Vat-PT method notably preserves most of the native characteristics observed in unprocessed human milk MFG. Regarding the lipolysis characteristics, homogenization increased the in vitro lipolysis of MFG, suggesting that this treatment could increase the digestibility of MFG. The findings from the present study provide an increased understanding of processing techniques’ effects on milk fat globules and milk fat globule membranes, which could be translated into clinical human milk research for alternative human milk processing techniques and their impact on infant health. 

## Figures and Tables

**Table 1:** MFG average diameter and zeta potential values of milk samples including Raw, Vat-PT, UHT, RTR, Raw-H, UHT-H, and H-RTR.

Samples	Diameter (μm)	Vol %	Width	Zeta Potential (mV)
Raw	13.55 ± 0.10	100	44.94 ± 1.32	−16.00 ± 0.50
Vat-PT	9.79 ± 0.38	100	38.83 ± 5.37	−16.50 ± 0.20
UHT	7.13 ± 0.34	100	11.55 ± 0.50	−24.60 ± 0.90
RTR	10.79 ± 0.78	100	27.76 ± 2.61	−20.10 ± 0.20
Raw-H	3.90 ± 0.04	100	7.74 ± 0.54	−23.10 ± 0.30
UHT-H	2.06 ± 0.44	100	4.86 ± 0.85	−24.40 ± 0.50
H-RTR	3.90 ± 1.40	100	9.41 ± 4.54	−21.20 ± 0.20

**Table 2:** L*, a* and b* values of milk samples, including raw, raw (thaw), Raw-H, Vat-PT, UHT, UHT-H, RTR, and H-RTR. One-way analysis of variance (ANOVA) with post hoc Tukey’s tests were used to identify significant differences between treatments, denoted by a star. ns: not significant, ****: *p* ≤ 0.0001.

Samples	L*	a*	b*
Raw	80.94 ± 0.06 ****	−4.15 ± 0.00 ****	9.76 ± 0.07 ****
Raw (thaw)	81.42 ± 0.04 ****	−3.89 ± 0.01 ****	10.16 ± 0.12 ****
Raw-H	86.72 ± 0.01 ****	−3.00 ± 0.00 ****	9.68 ± 0.01 ^ns^
Vat-PT	80.06 ± 0.04 ****	−3.66 ± 0.01 ****	8.64 ± 0.11 ****
UHT	82.71 ± 0.64 ****	−3.68 ± 0.00 ****	10.79 ± 0.63 ****
UHT-H	87.56 ± 0.25 ****	−3.00 ±0.00 ****	9.27 ± 0.13 ****
RTR	82.23 ± 2.81 ****	−2.29 ± 0.00 ****	15.07 ± 0.66 ****
H-RTR	85.11 ± 1.08 ****	−1.66 ± 0.04 ****	13.27 ± 0.37 ****

## Data Availability

The original contributions presented in the study are included in the article/Supplementary Materials, further inquiries can be directed to the corresponding authors.

## Supplementary Materials

The following supporting information can be downloaded at https://www.mdpi.com/article/10.3390/foods13081242/s1, Figure S1: Fluorescence microscopy images (×40 objective) and bright-field images of human milk, including Raw, Vat-PT, UHT, UHT-H, and Raw-H. Phospholipids were stained Oregon Green™ 488. Scale bars = 12 μm; Figure S2: The concentration of free sialic acid (Zoomed in) in human milk samples, including Raw, Raw-H, Vat-PT, UHT, UHT-H, RTR, and H-RTR. One-way analysis of variance (ANOVA) with post hoc Tukey’s test was used to identify significant differences between treatments, denoted by stars. * *p* ≤ 0.05.
