# Supplementary material for: Investigating Milk Fat Globule Structure, Size, and Functionality after Thermal Processing and Homogenization of Human Milk
Source: Foods. 2024 Apr 18;13(8):1242. doi: 10.3390/foods13081242 (PMC11049580; doi:10.3390/foods13081242)
Supplement: Supplementary file 1 [file foods-13-01242-s001.zip › foods-2911010-supplementary.pdf]

Supplementary Material

Investigating milk fat globule structure, size, and functionality after thermal processing and homogenization of human milk

Gulustan Ozturk[1,2*], Bruna Paviani[1], Rewa Rai[1], Randall C. Robinson[1], Sierra D. Durham[1], Mara I. Baller[1], Aidong Wang[1] Nitin Nitin[1], Daniela Barile[1,3*]

* Correspondence: Gulustan Ozturk, Daniela Barile; gozturk@wisc.edu, dbarile@ucdavis.edu

Supplementary Figures

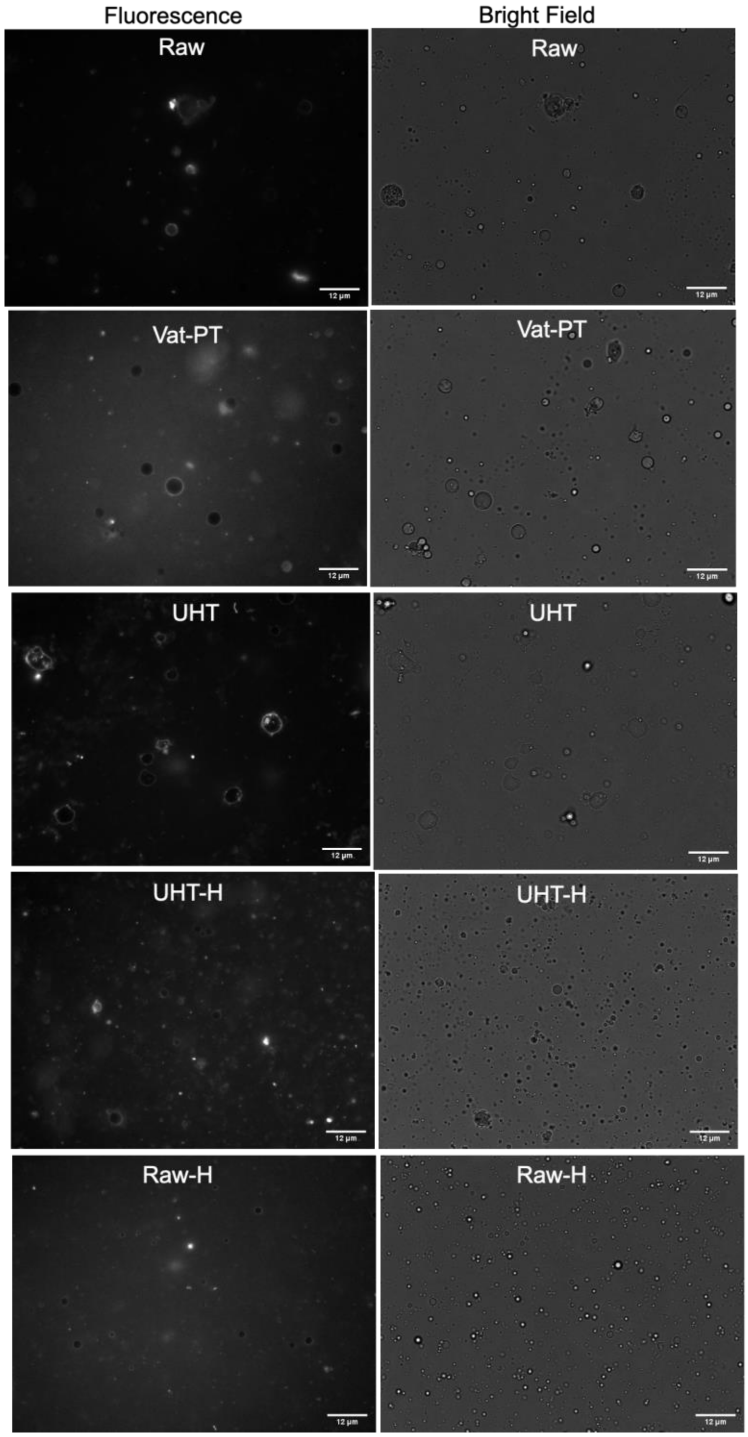

Figure S1. Fluorescence microscopy images (×40 zoom) and bright-field images of human milk, including Raw, Vat-PT, UHT, UHT-H, and Raw-H. Phospholipids were stained Oregon green. Scale bars = 12 μm.

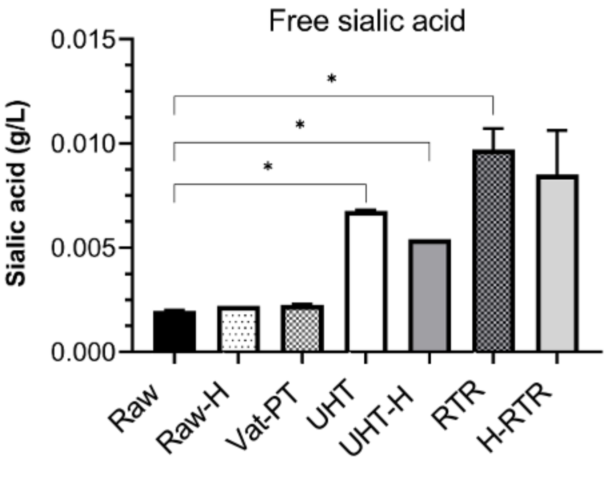

Figure S2. The concentration of free sialic acid (Zoomed in) in human milk samples, including Raw, Raw-H, Vat-PT, UHT, UHT-H, RTR, and H-RTR. One-way analysis of variance (ANOVA) with post hoc Tukey's test was used to identify significant differences between treatments, denoted by stars. *$P \leq 0.05$.
